# Supplementary material for: Enhanced NRT1.1/NPF6.3 expression in shoots improves growth under nitrogen deficiency stress in Arabidopsis
Source: Commun Biol. 2021 Feb 26;4:256. doi: 10.1038/s42003-021-01775-1 (PMC7910545; doi:10.1038/s42003-021-01775-1)
Supplement: Supplementary file 2 — Supplementary information [file 42003_2021_1775_MOESM2_ESM.pdf]

Supplementary inforamation

Enhanced *NRT1.1* expression in shoots improves growth under nitrogen deficiency stress in Arabidopsis

Yasuhito Sakuraba[1,†], Chaganzhana[1,†], Atsushi Mabuchi[2], Koh Iba[2], Shuichi Yanagisawa[1,*]

[1]Plant Functional Biotechnology, Biotechnology Research Center, The University of Tokyo, Bunkyo-ku, Tokyo 113-8657, Japan

[2]Department of Biology, Faculty of Science, Kyushu University, Fukuoka 819-0395, Japan

[†]These authors contributed equally to this work.

[*]Corresponding author:
Shuichi Yanagisawa
Tel: +81 3 5841 3066
E-mail: asyanagi@mail.ecc.u-tokyo.ac.jp

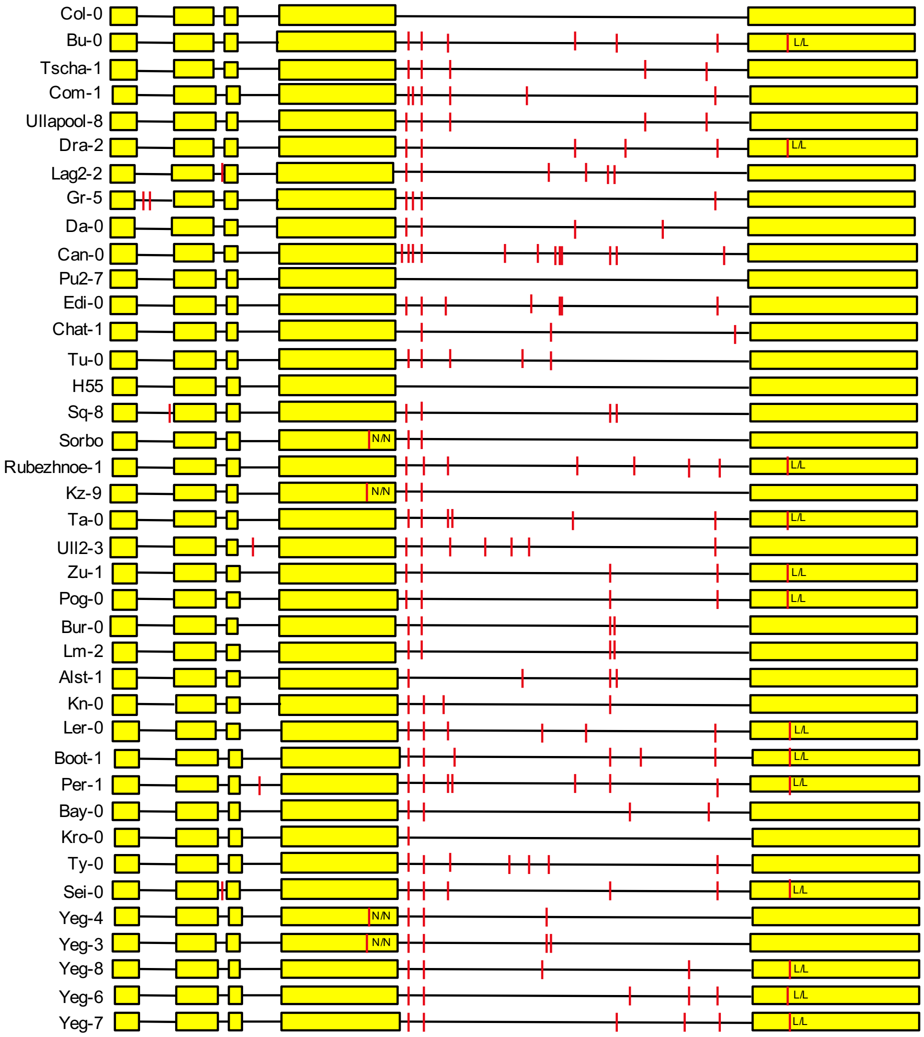

Supplementary Fig. 1 Polymorphisms among *NRT1.1* alleles of 39 Arabidopsis accessions. Information on polymorphisms in the *NRT1.1* locus of 39 Arabidopsis accessions was obtained from the Salk Arabidopsis 1,001 Genomes database (http://signal.salk.edu/atg1001/3.0/gebrowser.php). The gene structure of *NRT1.1* (from the start codon to the stop codon) is shown. Yellow boxes and black horizontal lines indicate exons and introns, respectively. Red vertical lines indicate positions of polymorphisms identified using the Col-0-type *NRT1.1* locus as the reference sequence. Polymorphisms detected in the 4th and 5th exons caused changes in codon usage but no amino acid substitutions (N/N and L/L, respectively, where N indicates asparagine, and L indicates leucine).

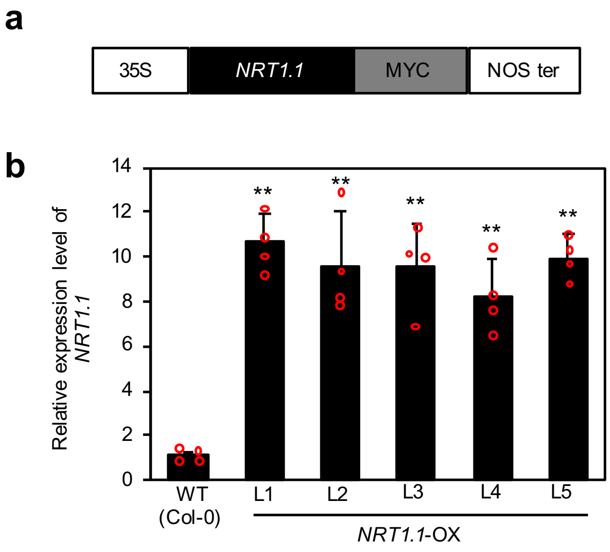

Supplementary Fig. 2 Expression levels of *NRT1.1* in five independent *NRT1.1*-OX lines. **a** Schematic representation of the construct used for the generation of *NRT1.1*-OX transgenic Arabidopsis plants. In the construct, Col-0-type *NRT1.1* cDNA in frame fused to the sequence encoding MYC epitope was cloned between the cauliflower mosaic virus *35S* promoter (35S) and nopaline synthase gene terminator (NOS ter). **b** RT-qPCR analysis of *NRT1.1* transcripts in five independent *NRT1.1*-OX transgenic lines (L1-L5) and wild-type Col-0 (WT) plants. Total RNA was isolated from 2-week-old seedlings grown on 1/2 MS agar plates. Primers used for RT-qPCR are listed in Supplementary Table 4. Data represent mean ± standard deviation (SD) of four biological replicates. Asterisks indicate significant differences between WT plants and other genotypes (**$p < 0.01$; Student's t-test).

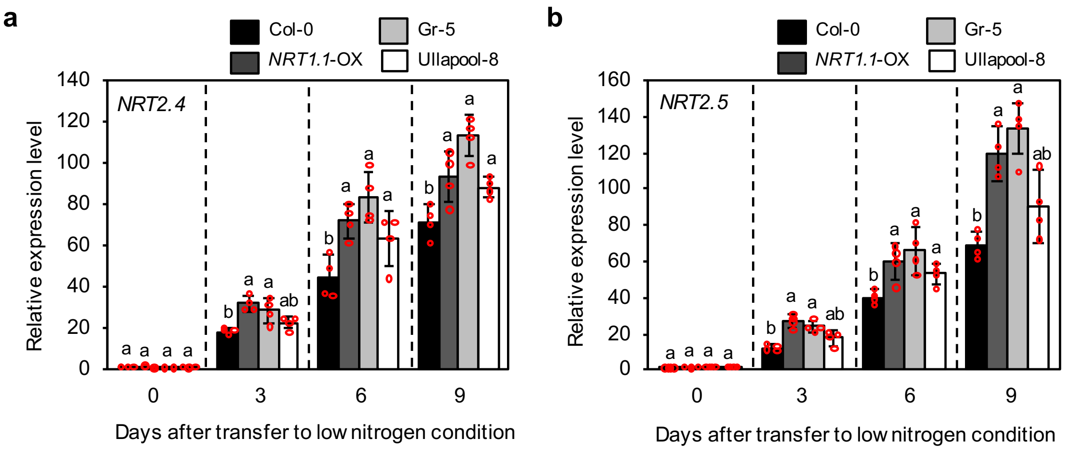

Supplementary Fig. 3 Expression levels of *NRT2.4* and *NRT2.5* in the shoots of Col-0, *NRT1.1*-OX, Gr-5, and Ullapool-8 seedlings. Seedlings were grown on 1/2 MS agar plates with continuous light for 5 days and then under the low N condition for the indicated duration. Transcript levels of *NRT2.4* (**a**) and *NRT2.5* (**b**) were normalised against transcript levels of *ACT2* and then against the value obtained from samples at time zero. Data represent mean ± SD of four biological replicates. Different lowercase letters indicate significant differences ($p < 0.05$; ANOVA followed by Tukey's post-hoc test).

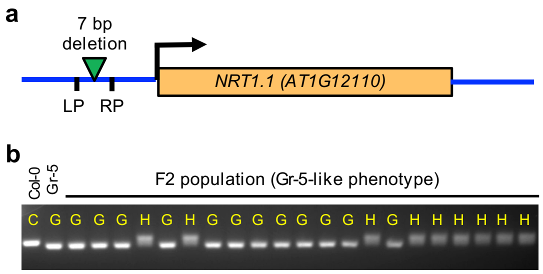

Supplementary Fig. 4 Genotyping the *NRT1.1* locus in Col-0 × Gr-5 F2 progeny by PCR. **a** Schematic representation of the *NRT1.1* locus. The location of a 7 bp deletion found in the Gr-5-type *NRT1.1* promoter is indicated by a green inverted triangle, and the positions of primers (LP and RP) used for genotyping the *NRT1.1* locus are indicated. **b** An example of PCR-based genotyping of the F2 progeny of the Col-0 × Gr-5 cross. C, individuals homozygous for the Col-0-type *NRT1.1* allele; G, individuals homozygous for the Gr-5-type *NRT1.1* allele; H, heterozygous individuals.

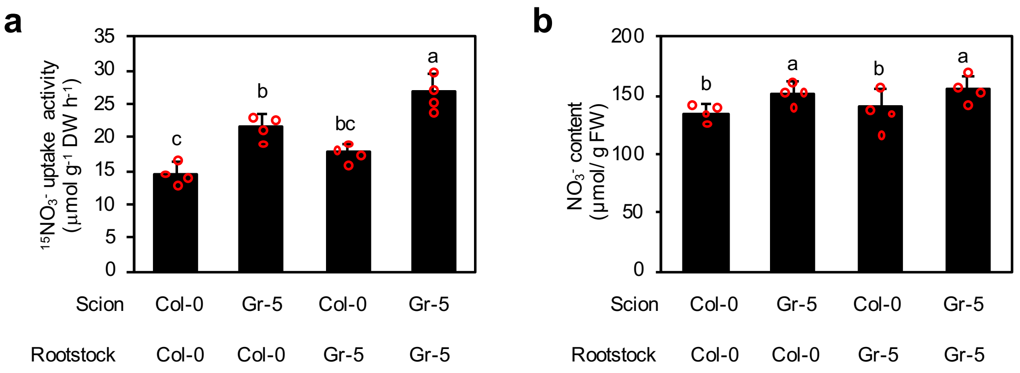

Supplementary Fig. 5 Uptake and concentration of nitrate in grafted Arabidopsis seedlings. **a** Uptake of ^{15}N-labeled nitrate by grafted seedlings. Grafted seedlings were generated using the aerial and subterranean parts of 5-day-old Col-0 and Gr-5 seedlings grown with continuous light as scions and rootstocks in the indicated combinations, and were then grown on 1/2 MS agar plates for 4 days. Data represent mean ± SD of four biological replicates. **b** Nitrate concentrations in grafted seedlings. Data represent mean ± SD of five biological replicates. In **a** and **b**, different lowercase letters indicate significant differences ($p < 0.05$; ANOVA followed by Tukey's post hoc test).

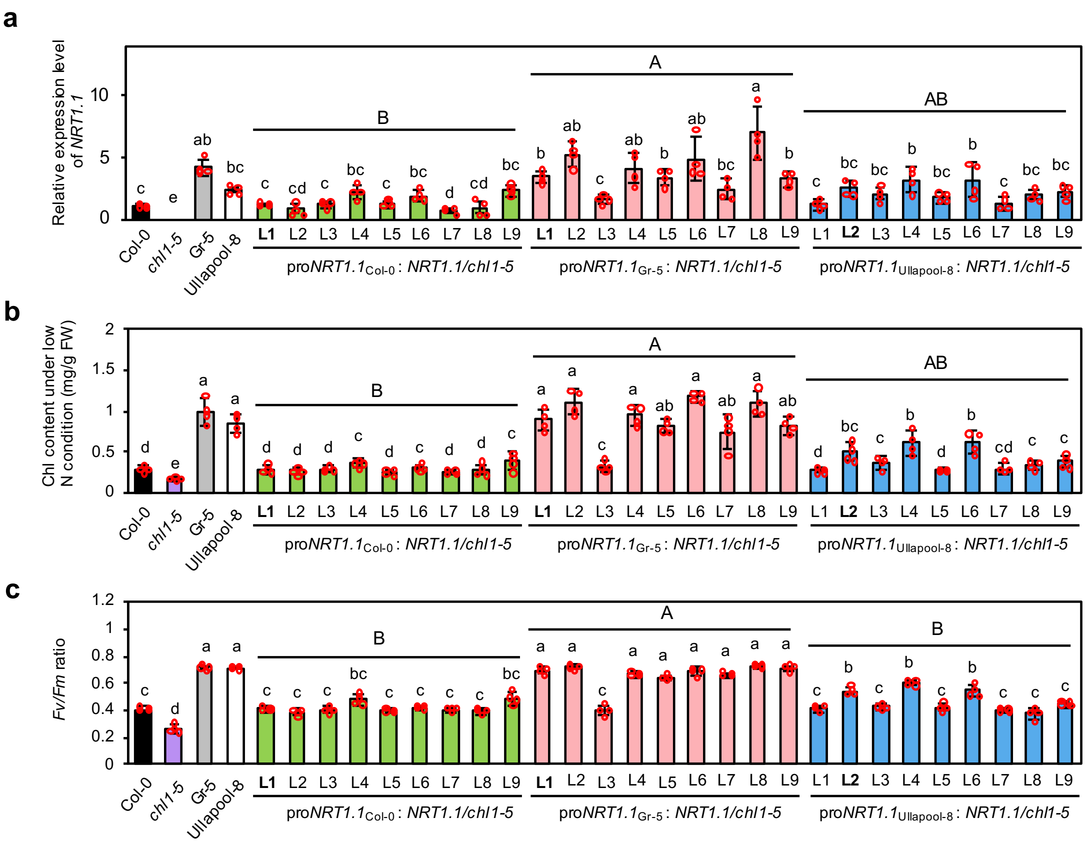

Supplementary Fig. 6 Characterization of transgenic lines expressing Col-0-type *NRT1.1* cDNA under the control of the Col-0-, Gr-5-, or Ullapool-8-type *NRT1.1* promoter in the *chl1-5* mutant background. a Levels of *NRT1.1* transcripts in the shoots of 10-day-old seedlings grown on 1/2 MS agar plates. Transcript levels of *NRT1.1* were normalised relative to those of *ACT2* and then against the value obtained from Col-0 samples. **b**, **c** Total chlorophyll contents (**b**) and *Fv/Fm* ratios (**c**) of the shoots of seedlings grown for 5 days on 1/2 MS agar plates with continuous light and then for 5 days under the low N condition (0.03 mM N). In **a**–**c**, nine independent lines generated for each of the three constructs (pro*NRT1.1*$_{\text{Col-0}}$:*NRT1.1/chl1-5*, pro*NRT1.1*$_{\text{Gr-5}}$:*NRT1.1/chl1-5*, and pro*NRT1.1*$_{\text{Ullapool-8}}$:*NRT1.1/chl1-5*) were analysed, together with Col-0, *chl1-5*, GR-5 and Ullapool-8 seedlings. Data shown in **a**, **b**, and **c** were also used for Fig 6a, 6c, and 6d. Data represent mean ± SD of four biological replicates. Different lowercase letters indicate significant differences among all lines used in the experiments ($p < 0.05$; ANOVA followed by Tukey's post-hoc test). Significant differences among effects generated by different constructs were also analysed using mean ± SD of the values obtained with nine independent transgenic lines for each of the construct. Different uppercase letters shown above lowercase letters indicate significant differences among the effects caused by different constructs ($p < 0.05$, ANOVA followed by Tukey's post hoc test).

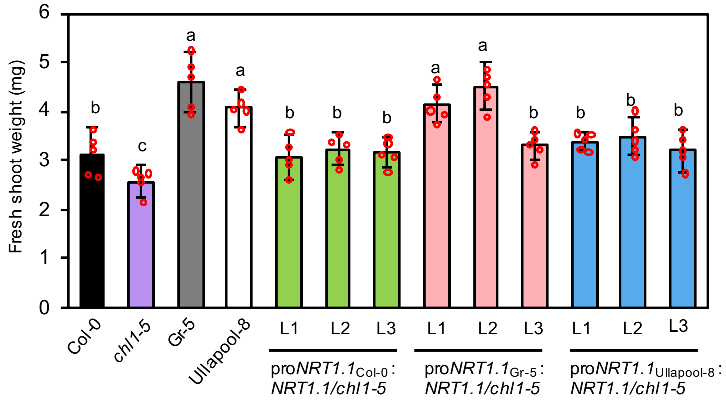

Supplementary Fig. 7 Effect of Col-0-, Gr-5-, and Ullapool-8-type *NRT1.1* promoter-driven *NRT1.1* expression on the growth of the *nrt1.1* null mutant (*chl1-5*). Fresh shoot weights of Col-0, *chl1-5*, Gr-5, Ullapool-8, and pro*NRT1.1*$_{\text{Col-0}}$:*NRT1.1/chl1-5* L1–3, pro*NRT1.1*$_{\text{Gr-5}}$:*NRT1.1/chl1-5* L1–3, and pro*NRT1.1*$_{\text{Ullapool-8}}$:*NRT1.1/chl1-5* L1–3 seedlings are shown. Seedlings were grown for 5 days on 1/2 MS agar plates with continuous light and then for 5 days under the low N condition (0.03 mM N). Data represent mean ± SD of five biological replicates. Different lowercase letters indicate significant differences (p < 0.05; ANOVA followed by Tukey's post-hoc test).

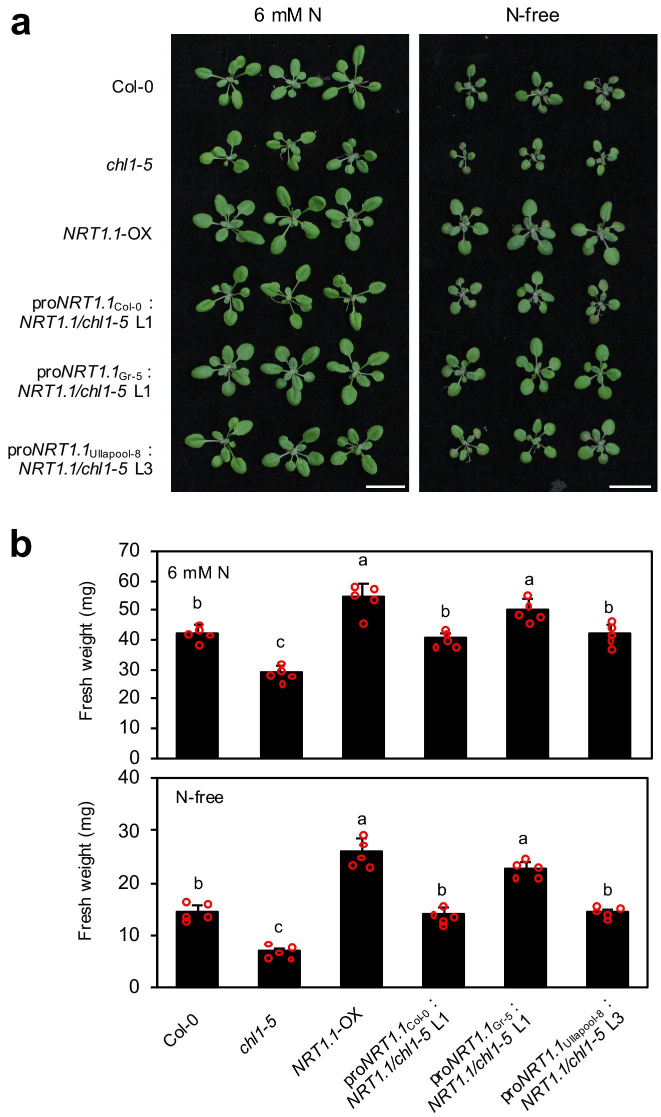

Supplementary Fig. 8 Modulation of N deficiency-induced growth inhibition of *chl1-5* mutant plants by the expression of *NRT1.1* under the control of the *35S* promoter or the Col-0-, Gr-5-, or Ullapool-8-type *NRT1.1* promoter. a, **b** Images (**a**) and fresh shoot weight (**b**) of Col-0, *chl1-5*, *NRT1.1*-OX L1, pro*NRT1.1*$_{\mathrm{Col\text{-}0}}$:*NRT1.1/chl1-5* L1, pro*NRT1.1*$_{\mathrm{Gr\text{-}5}}$:*NRT1.1/chl1-5* L1, and pro*NRT1.1*$_{\mathrm{Ullapool\text{-}8}}$:*NRT1.1/chl1-5* L3 plants initially grown hydroponically in 6 mM N-containing medium for 10 days and further grown in 6 mM N-containing medium or N-free medium for 14 days under continuous light condition. Data represent mean ± SD of six biological replicates. Different lowercase letters indicate significant differences ($p < 0.05$; ANOVA followed by Tukey's post-hoc test). Scale bar = 5 cm.

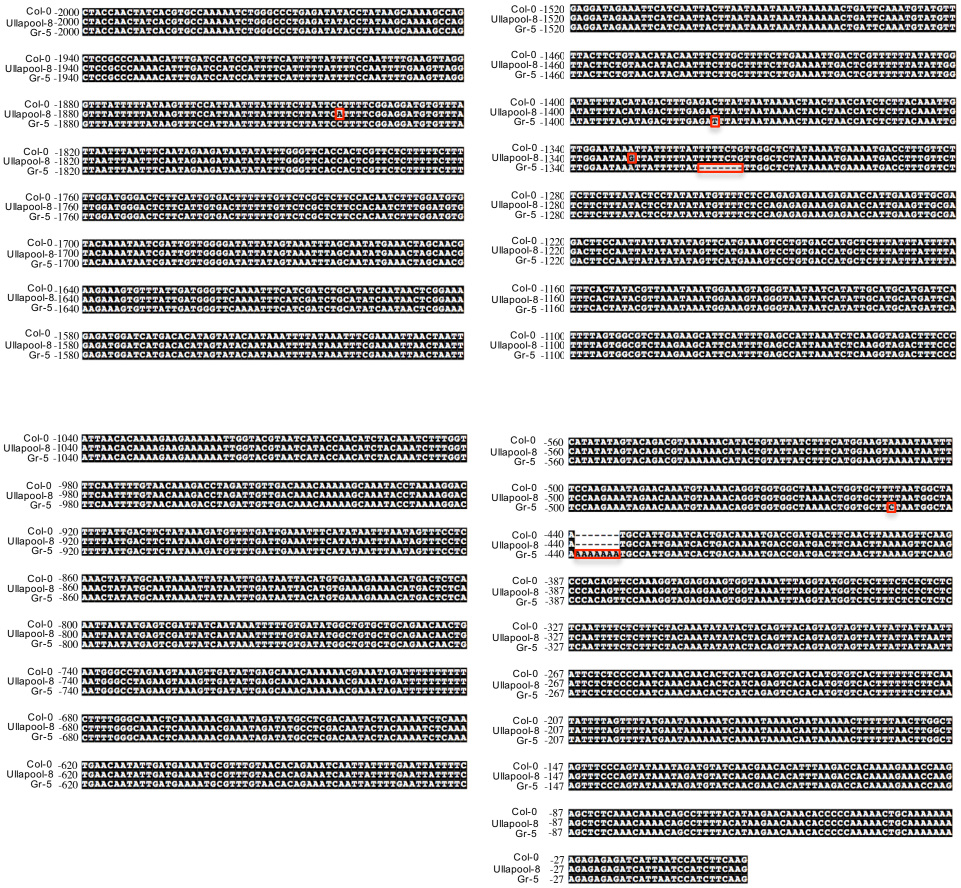

Supplementary Fig. 9 Nucleotide sequence alignment of the Col-0-, Gr-5-, and Ullapool-8-type *NRT1.1* promoters. Two kilobase pairs of the *NRT1.1* promoter sequence (relative to the translational start site) of Col-0, Gr-5, and Ullapool-8 are shown. The numbers indicated are based on the Col-0-type *NRT1.1* promoter sequence. Multiple sequence alignment was performed using Clustal Omega (https://www.genome.jp/tools-bin/clustalw), and the alignment file was shaded using the BoxShade program (https://embnet.vital-it.ch/software/BOX_form.html). Conserved regions are shaded in black, and polymorphisms are outlined with red boxes.

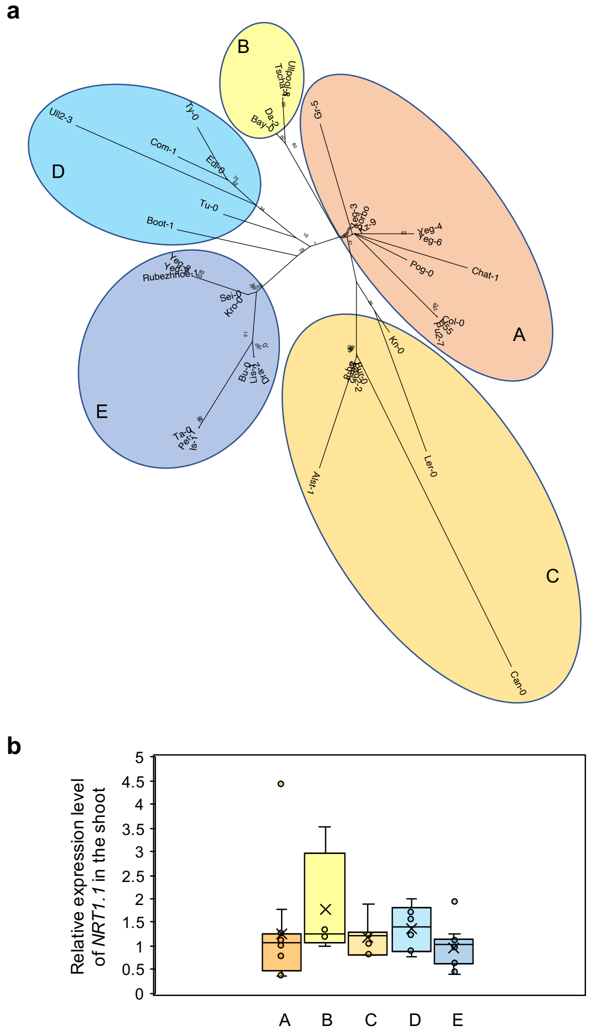

Supplementary Fig. 10 Haplogroups identified using 4th intron sequences of *NRT1.1*. Information on polymorphisms in the 4th intron of *NRT1.1* of 39 Arabidopsis accessions was obtained from the Salk Arabidopsis 1,001 Genomes database (http://signal.salk.edu/atg1001/3.0/gebrowser.php). **a.** A haplogroup tree constructed using the MEGA X software by the neighbor-joining method with 1000 bootstrap replicates. Haplotypes at the *NRT1.1* locus were classified into five haplogroups (A-E). **b**. A box-plot diagram of the relative expression level of *NRT1.1* in accessions of which haplotypes were classified into different haplogroups. There is no significant difference in the expression levels among

accessions with haplotypes that belong to different haplogroups ($p < 0.05$; Tukey's multiple comparison test). The outlier in haplogroup A is derived from Gr-5.

Supplementary Table 1 Results of PCR-based genotyping of the *NRT1.1* promoter allele in the F2 progeny of the Col-0 × Gr-5 cross[a].

Experiment	Total no. of plants analyzed	No. of plants homozygous for the Col-0 allele	No. of heterozygous plants	No. of plants homozygous for the Gr-5 allele
Col-0-like				
Experiment 1	85	73	12	0
Experiment 2	83	69	14	0
Gr-5-like				
Experiment 1	82	0	54	28
Experiment 2	91	0	58	33

[a]In each experiment in which more than 300 seedlings were grown under the low N conditions.

We note that in each experiment, the ratio of the number of Col-0-like F2 seedlings to the number of Gr-5-like F2 seedlings was similar to the result shown in Table 1. Genotyping was performed using all Col-0-like seedlings and randomly selected Gr-5-like F2 seedlings.

Supplementary Table 2 Polymorphisms in the Gr-5- and Ullapool-8-type *NRT1.1* promoters.

Accession	Polymorphism	Position (bp)[a]	Transcription factor-binding motif
Ullapool-8	C to A	-1,840	Dof binding motif (CTTTT)
Ullapool-8	A to G	-1,331	AT-Hook binding motif (ATAAA)
Gr-5	7 bp deletion	-1,319 to -1,313	RAV binding motif (TGTTG)
Gr-5	7 bp insertion	-443 to -437	AT-Hook binding motif (ATAAA)

[a]Numbers indicate nucleotide positions relative to the translational start site in the Col-0-type *NRT1.1* promoter.

Supplementary Table 3 List of PCR primers used in this study.

Target DNA	Forward primer sequence (5'→3')	Reverse primer sequence (5'→3')
Plasmid construction		
NRT1.1 promoter (2.0 kb)	GTGGTCCTACCAACTATCACGT	CTTGAAGATGGATTAATGATCTCT
NRT1.1 coding sequence	ATGTCTCTTCCTGAAACTAAATC	TCAATGACCCATTGGAATACTCG
Construction of reporter plasmids		
NRT1.1 promoter	AAACTGCAGCTACCAACTATCA	AAAGTCGACCTTGAAGATGGATT
NRT1.1 promoter P1	AAACTGCAGCTACCAACTATCA	ATAGAGCCAAATAAAAATAATTT ATTCC<br>AAAGTCGACCTTGAAGATGGATT
NRT1.1 promoter P2	TTAATGGCTAAAAAAAAATGCC ATTGAAT<br>AAACTGCAGCTACCAACTATCA	AAAGTCGACCTTGAAGATGGATT
NRT1.1 promoter P3	AAACTGCAGCTACCAACTATCA	CCGAAAATGAATAAGAAAATAAA TTAA<br>AAAGTCGACCTTGAAGATGGATT
NRT1.1 promoter P4	AAACTGCAGCTACCAACTATCA	AATAAAAATAACTTATTCCAACA ATTTG<br>AAAGTCGACCTTGAAGATGGATT
Genotyping Col-0 × Gr-5 F2 progeny		
NRT1.1 allele	CTAACTAACCATCTCTTACAAAT TGTTG	TAAAGAGCATGGTCACAGGACT
RT-qPCR		
ACT2	TGGGATGAACCAGAAGGATG	AAGAATACCTCTCTTGGATTGTGC
NRT1.1	AAGGTATGAAAGGGATGAGCA	TCGACGATTGTCACGAGAAC
NRT2.1	TGAGCAGGAGAAGCAGAAGA	TTGTTGGGTGTGTTCTGAGG
SGR1	TGGGCAAATAGGCTATACCG	CCACCGCTTATGTGACAATG
