# Supplementary material for: Enhanced NRT1.1/NPF6.3 expression in shoots improves growth under nitrogen deficiency stress in Arabidopsis
Source: Commun Biol. 2021 Feb 26;4:256. doi: 10.1038/s42003-021-01775-1 (PMC7910545; doi:10.1038/s42003-021-01775-1)
Supplement: Supplementary file 3 — Description of Additional Supplementary Files [file 42003_2021_1775_MOESM3_ESM.pdf]

Description of Additional Supplementary Files

File name: Supplementary Data 1

Description: List of *Arabidopsis thaliana* accessions used in this study, along with their chlorophyll (Chl) contents under the control N conditions (6 mM N; $Chl_{control}$) and low N condition ($Chl_{low\ N}$). Data represent mean ± S.D. of five biological replicates.

File name: Supplementary Data 2

Description: Summary statistics for GWAS shown in Fig. 1c. The data was obtained from easyGWAS web interface ((https://easygwas.ethz.ch).

File name: Supplementary Data 3

Description: Raw source data used to create figures in main text and Supplementary information.
